# Comparison of Time Domain and Frequency-Wavenumber Domain Ultrasonic Array Imaging Algorithms for Non-Destructive Evaluation

**DOI:** 10.3390/s20174951

**Published:** 2020-09-01

**Authors:** Zeyu Zhuang, Jie Zhang, Guoxuan Lian, Bruce W. Drinkwater

**Affiliations:** 1 State Key Laboratory of Acoustics, Institute of Acoustics, Chinese Academy of Sciences, Beijing 100190, China; lian@mail.ioa.ac.cn; 2 University of Chinese Academy of Sciences, Beijing 100190, China; 3 Department of Mechanical Engineering, University of Bristol, Bristol BS8 1TR, UK; j.zhang@bristol.ac.uk (J.Z.); b.drinkwater@bristol.ac.uk (B.W.D.)

**Keywords:** ultrasonic arrays, non-destructive testing, plane wave imaging, total focusing method, frequency-wavenumber migration

## Abstract

Ultrasonic array imaging algorithms have been widely developed and used for non-destructive evaluation (NDE) in the last two decades. In this paper two widely used time domain algorithms are compared with two emerging frequency domain algorithms in terms of imaging performance and computational speed. The time domain algorithms explored here are the total focusing method (TFM) and plane wave imaging (PWI) and the frequency domain algorithms are the wavenumber algorithm and Lu’s frequency-wavenumber domain implementation of PWI. In order to make a fair comparison, each algorithm was first investigated to choose imaging parameters leading to overall good imaging resolution and signal-to-noise-ratio. To reflect the diversity of samples encountered in NDE, the comparison is made using both a low noise material (aluminium) and a high noise material (copper). It is shown that whilst wavenumber and frequency domain PWI imaging algorithms can lead to fast imaging, they require careful selection of imaging parameters.

## 1. Introduction

Ultrasonic arrays have been widely used for non-destructive evaluation (NDE) in recent years [1]. Ultrasonic arrays have advantages over single-element devices such as the ability of one array to produce beams at a range of angles or focal depths, and the intuitive imaging of the interior of a structure that they produce as an output. In array imaging the overall approach, independent of imaging algorithm is to: (1) fire the array elements in some sequence to generate an ultrasound beam in the structure, (2) the reflected ultrasound signals are captured by the same array elements and (3) these received signals are processed to form the image.

This paper discusses two categories of imaging algorithm, time domain and frequency-wavenumber (f-k) domain methods. In the time domain, or delay-and-sum (DAS) algorithms [2], the amplitude of an image pixel is the summation of the amplitudes of the received signals after the application of time delays, typically set to represent a specific wave propagation path. These imaging algorithms are relatively simple to implement and can be configured for a wide variety of inspection cases. Among the possible time domain imaging algorithms, the total focusing method (TFM) [3] and plane wave imaging (PWI) [4] are the focus of this study. The TFM imaging algorithm requires full matrix capture (FMC) in which all transmit-receive signals are captured and hence an N-element array requires N transmission cycles. All the transmitter–receiver pairs are used in the summation to produce a synthetic focus at each pixel. Variants of the approach have been used to inspect structures with multiple layers [5], through nonplanar surfaces [6] and considering wave mode conversions at a defect or an interface [7]. Because the time domain approaches are based on a simple summation, the algorithms are readily parallelisable using graphics processing units (GPU) and field-programmable gate arrays (FPGA). Such approaches have been used for fast TFM imaging [8,9] and can achieve imaging speeds of 150 frames/s for an image with 200 × 200 pixels and a 32 element array [9].

The time domain PWI algorithm uses a discrete number of plane waves with various steering angles to inspect a structure [4]. Relative to the FMC, PWI typically results in a smaller number of transmit cycles and, as the energy associated with each transmitted plane wave is greater than that of an individual element emission, larger penetration depths can be achieved. By optimising the steering angles of the transmitted plane waves, this method can achieve a high frame rate with adequate image quality. The PWI algorithm has also been developed for multimodal imaging cases [10] and inspecting the structures through an irregular surface [11].

The development of f-k domain imaging algorithms was motivated by fast image formation, capitalising on the efficiency of the Fourier transform algorithm and a reduced number of computations. In general, these algorithms transform the array data into the frequency-wavenumber (f-k) domain where a mapping/migration operation is performed, followed by an inverse Fourier transform to the image domain. Stolt [12] first derived what is now known as Stolt’s f-k migration algorithm for applications in seismology. After that, the f-k domain imaging algorithm has been developed in both the medical imaging and NDE fields. In medical ultrasound imaging field, Lu et al. [13] proposed a method that uses a single transmitted plane wave to achieve very high imaging frame rate and demonstrated 3750 frames/s in 200 mm depth biological tissues. Chen et al. [14] improved image resolution by extending this to use multiple plane waves at different angles. Garcia et al. [15] applied Stolt’s method to PWI by modifying the exploding reflector model and achieved good contrast-to-noise ratio and lateral image resolution. Inspired by medical ultrasound imaging, Stepinski et al. [16] implemented the f-k imaging algorithm in the NDE field and showed good results could be achieved on typical test structures. Skjelvareid et al. [17] used the f-k domain imaging algorithm to inspect multi-layered structures. Hunter et al. [18] proposed the wavenumber imaging algorithm which uses an FMC array data set to achieve a high image resolution and increase the image speed by a factor of the number of array elements compared to the TFM imaging algorithm. More recently, Moghimirad et al. [19] developed a frequency domain algorithm that generates focused virtual sources and this achieved large penetration depth and 20 times faster imaging speed than the time domain equivalent.

Velichko et al. [20] theoretically analysed the connections between the TFM and wavenumber imaging algorithms by expressing them as a linear superposition of phase-delayed transmitter–receiver signals in the frequency domain with different focusing coefficients. They demonstrated that the wavenumber method can provide lower side lobes in the image than the TFM as the transformation process acts as a frequency-independent spatial filter. In terms of computational performance, although the wavenumber algorithm is superior, it requires a large amount of computational memory. Zhang et al. [21] and Anton et al. [22] proposed methodologies to assess the suitability of ultrasound imaging algorithms for NDE, especially for inspecting materials with large grains which can cause strong ultrasound backscattering that results in noise in ultrasound images. A general conclusion is that the TFM imaging algorithm is the most robust algorithm with largest flexibility for inspecting a range of different types of defects [21,22]. Merabet et al. [23] theoretically compared the time domain and f-k domain PWI and concluded that, for computational performance implemented using Matlab, the f-k method is faster than time domain PWI both in 2D and 3D imaging. In terms of image quality, the f-k method improves the lateral resolution because of the spatial-frequency filter effect mentioned above. It is noted that all above comparisons were based on defects at a specific location (generally direct below the array centre). Hence, whilst these papers give insights to imaging algorithm performance in specific NDE inspection examples, they do not present a general picture of the algorithm performance on the diverse range of inspection scenarios encountered.

The motivation of this paper is to comprehensively compare the most commonly used ultrasound imaging algorithms in NDE field with respect to the performance on defects at different locations and in materials with different backscattering noise. Hence, this paper represents a useful guide to imaging performance and how to choose and optimise these imaging algorithms in practice. It is hoped that this represents a further step towards the industrial uptake of these imaging techniques. The algorithms are first overviewed, the methodology of how to choose imaging parameters for time domain and f-k domain PWI algorithms is then proposed, the chosen imaging parameters are finally used to form images for the comparison. Note that given their widespread industrial application, this paper is limited to the application of 1D linear arrays for 2-D imaging on a 2-D structure.

## 2. Overview of Ultrasound Imaging Algorithms Compared

In this section, the four chosen imaging algorithms are briefly overviewed. The TFM imaging algorithm and the time domain PWI algorithm are implemented by mapping the delayed and summed time domain signals to the image domain directly. In the wavenumber imaging algorithm and the f-k domain PWI algorithm, the time domain signals are first transformed to the f-k domain using a Fourier transform (FT), the different mapping methods are then applied in the f-k domain and the final image is generated using an inverse Fourier transform (IFT).

**Figure 1:**
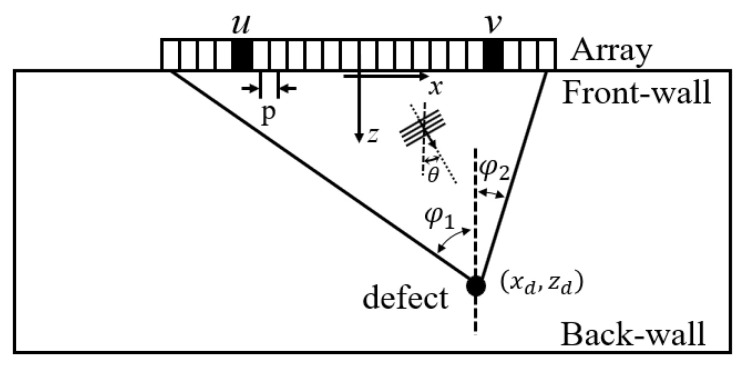
Schematic diagram illustrating the array and sample geometry used in both the experiments and simulations.

Figure 1 shows the 1-D array and 2-D test structure geometry as well as the coordinate system used in both the experimentally measured and simulated images. In an FMC data set captured by a 1-D linear array with $N_{e}$ elements from a 2-D structure, e(u,v,t) represents the time domain signal transmitted by the element at (u,0) and received by an array element at (v,0). In a plane wave data set with $N_{p}$ transmitted plane waves, f(θ,v,t) represents the time domain signal transmitted with a plane wave steering angle of θ and received by an array element at (v,0). In the simulation presented in this paper, we synthesize the plane waves from the FMC data set by (1),

(1) $$f\left(\theta ,v,t\right)=\sum_{u}e\left(u,v,t-u\sin\theta /c\right)$$

where, *c* is the longitudinal wave speed in the test structure material.

### 2.1. TFM Imaging Algorithm

The intensity of the TFM image at pixel located at (x,z) is summation of the appropriately delayed time domain data across all transmitter–receiver combinations and can be expressed as [3],

(2) $$I_{\mathrm{TFM}}\left(x,z\right)=\left|\sum_{u}\sum_{v}e\left(u,v,\tau \left(u,x,z\right)+\tau \left(v,x,z\right)\right)\right|$$

where, τ is the wave propagation time from an array element to a pixel at (x,z) and,

(3) $$\begin{array}{c} \tau \left(u,x,z\right)=\frac{d(u,x,z)}{c}=\frac{\sqrt{{\left(x-u\right)}^{2}+z^{2}}}{c},\\ \tau \left(v,x,z\right)=\frac{d(v,x,z)}{c}=\frac{\sqrt{{\left(x-v\right)}^{2}+z^{2}}}{c} \end{array}$$

where, *d* is the distance from an array element to a point at (x,z).

### 2.2. Wavenumber Imaging Algorithm

In forming a wavenumber image, the f-k domain data set, $E(k_{u},k_{v},\omega )$, is first generated from an FMC array data set using a 3D FT by [18],

(4) $$E\left(k_{u},k_{v},\omega \right)=\;\int \int \int \;e\left(u,v,t\right)e^{-i(\omega t+k_{u}u+k_{v}v)}dtdudv$$

where, ω is the angular frequency, $k_{u}$ and $k_{v}$ are the wavenumbers in the f-k domain described in more detail in Section 4.1. $E(k_{u},k_{v},\omega )$ is then mapped into the image Fourier domain to generate the Fourier spectrum at each $k_{u}$ using an inverse Stolt mapping. The Fourier spectrum of the final image is the summation of the Fourier spectra at all $k_{u}$, expressed as [12],

(5) $$\begin{array}{c} S\left(k_{x},k_{z}\right)=\sum_{k_{u}}\left\{S^{-1}\left[-\left(4\pi^{2}\right)\sqrt{k^{2}-{k_{u}}^{2}}\sqrt{k^{2}-{k_{v}}^{2}}E\left(k_{v},\omega |k_{u}\right)\right]\right\} \end{array}$$

where, $S^{-1}\left[\cdot \right]$ denotes the inverse Stolt mapping, $k_{x}$ and $k_{z}$ are the wavenumbers in the image Fourier and are described in more detail in Section 4.1. The relationship between the wavenumbers in the f-k domain and the image Fourier domain is [12],

(6) $$k_{v}=k_{x}-k_{u},k_{z}=\sqrt{k^{2}-{k_{u}}^{2}}+\sqrt{k^{2}-{k_{v}}^{2}}$$

and this leads to,

(7) $$k=\frac{\pm {\left({k_{z}}^{4}+2\left({k_{u}}^{2}+{k_{v}}^{2}\right){k_{z}}^{2}\right)+{k_{u}}^{4}+{k_{v}}^{4}-2{k_{u}}^{2}{k_{v}}^{2})}^{\frac{1}{2}}}{2k_{z}}$$

The intensity of the final image is the 2D IFT of $S(k_{x},k_{z})$ expressed as,

(8) $$I_{\mathrm{wn}}\left(x,z\right)=\left|{F_{k_{x},k_{z}}}^{-1}\left\{S\left(k_{x},k_{z}\right)\right\}\right|$$

### 2.3. Time Domain PWI Algorithm

In the time domain PWI algorithm, the image intensity at a pixel located at (x,z) is the summation of the images from a number of plane waves with different steering angles [4],

(9) $$I_{t-\mathrm{PWI}}\left(x,z\right)=\left|\sum_{\theta }\sum_{v}e\left(\theta ,v,\tau \left(\theta ,x,z\right)+\tau \left(v,x,z\right)\right)\right|$$

where, τ is the wave propagation time from an array element to a pixel at (x,z) and,

(10) $$\tau \left(\theta ,x,z\right)=\frac{x\sin\theta +z\cos\theta }{c}$$

### 2.4. Lu’s f-k Domain PWI Algorithm

In Lu’s f-k domain PWI algorithm [13], the spectrum of f(θ,v,t) is first calculated using a 2D FT with respect to *v* and *t*, as,

(11) $$F\left(\theta ,k_{v},\omega \right)=\;\int \int \;f\left(\theta ,v,t\right)e^{-i(\omega t+k_{v}v)}dtdv$$

$F(\theta ,k_{v},\omega )$ is then remapped in the image Fourier domain to generate the spectrum of the image using the inverse Stolt mapping,

(12) $$S\left(k_{x},k_{z}\right)=\sum_{\theta }\left\{S^{-1}\left[\sqrt{k^{2}-{k_{v}}^{2}}F\left(\theta ,k_{v},\omega \right)\right]\right\}$$

where,

(13) $$k_{v}=k_{x}-k\sin\theta ,k=\frac{{k_{x}}^{2}+{k_{z}}^{2}}{2k_{x}\sin\theta +k_{z}\cos\theta }$$

The intensity of the final image is the 2D IFT of the summation of all $S(\theta ,k_{x},k_{z})$ over $k_{x}$ and $k_{z}$,

(14) $$I_{\mathrm{fk}-\mathrm{PWI}}\left(x,z\right)=\left|{F_{k_{x},k_{z}}}^{-1}\sum_{\theta }\left\{S(\theta ,k_{x},k_{z})\right\}\right|$$

## 3. Experimental Setup and Simulation Model

### 3.1. Experimental Setup

**Figure 2:**
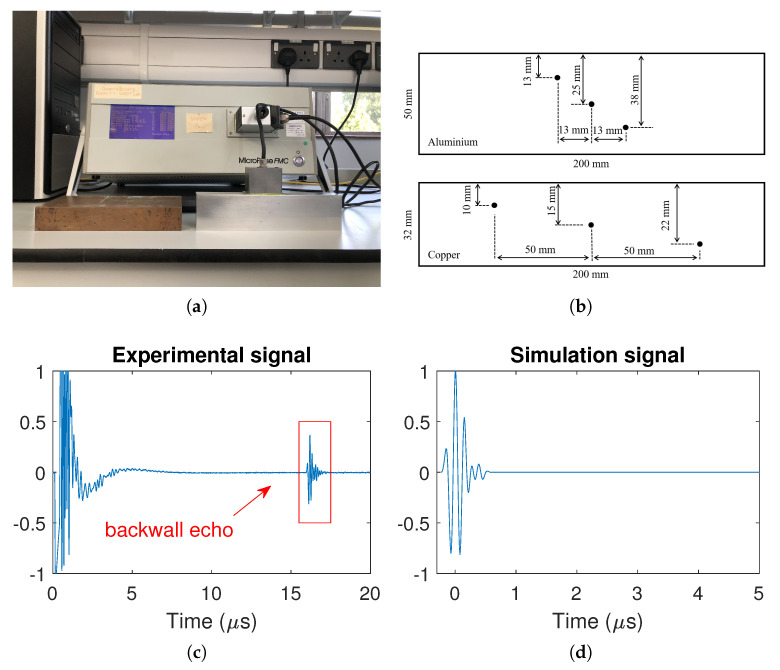
(**a**) Test rig and instrumentation, (**b**) specimen geometry and defect details, (**c**) an example of the experimentally measured pulse-echo signal from a typical array element and (**d**) the back-propagated signal of the back-wall reflection. Note that (**d**) is also the transmitted signal used in the simulation.

A 5-MHz linear array with 64 elements (manufactured by Imasonic, Besancon, France), of parameters shown in Table 1, was used in both the experiments and simulations. The array probe was coupled (using Sonagel-W250, Sonatest Ltd., Milton Keynes, UK) to the top surface of the specimens as shown in Figure 1, its *x*-location adjusted depending on the defect under test, and an FMC captured. A commercial array controller (Micropulse MP5PA, Peak NDT Ltd., Derby, UK) was used to capture the complete set of time domain signals from every transmitter–receiver pair of the ultrasonic array (i.e., the FMC data set), as shown in Figure 2a. The excitation signal from the array controller is a negative square wave with a pulse width of 80 ns, which leads to the frequency ranging from 0 to 12.4 MHz. The captured data were then exported and processed using MATLAB (The MathWorks Inc., Natick, MA, USA). The experimental data used in this paper are openly available in Supplementary Materials.

Figure 2b shows the details of specimens’ geometry and defect locations. An aluminium specimen of 50 mm thick in *z* direction was used for the low backscattering noise case and a copper specimen of 32 mm thick was used for the high backscattering noise case. The aluminium specimen had three well-spaced 1-mm-diameter side-drill-holes (SDHs) at *z* = 13, 25 and 38 mm and the copper specimen had three well-spaced 2-mm-diameter SDHs at *z* = 10, 15 and 22 mm. Note that the data sets used for generating plane wave images were synthesized from the FMC data using (1). Figure 2c shows an example of the experimentally measured pulse-echo signal from an array element, in which the signal reflected from the back-wall is labelled. The back-propagated version of this signal is shown in Figure 2d.

In the image from a defect (either simulated or experimentally measured), the array performance indicator (API) is a measure of the size of the point spread function and hence relates to image resolution [3],

(15) $$\mathrm{API}=N_{pi}\Delta x\Delta z/{\lambda_{c}}^{2}$$

where, $N_{pi}$ is the number of pixels with an amplitude above some threshold (e.g., −6 dB relative to the peak amplitude at the defect), Δx and Δz are pixel sizes and $\lambda_{c}$ is the wavelength at the centre frequency.

The signal to noise ratio (SNR) governs the detectability of a defect and was calculated from,

(16) $$\mathrm{SNR}=I_{d}/rms(I_{f})$$

where, $I_{d}$ is the peak amplitude from the image with a defect present and $rms(I_{f})$ is the root-mean-squared (RMS) of a region of the image (e.g., a 5 × 5 mm square box) centred on the defect location, but with the array placed over a region without a defect.

### 3.2. Simulation Model

**Figure 3:**
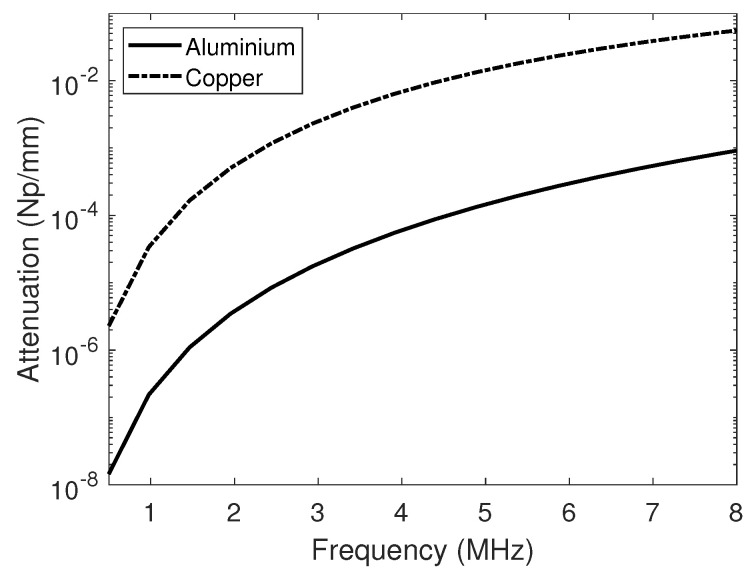
Experimentally measured longitudinal wave attenuation from the aluminium and copper samples used in the experiments.

A 2-D linear systems modelling approach [21,24] is used to simulate the FMC data set from a defect and hence produce noise-free images for comparison purposes. In the frequency domain, for a wave path from transmitter element at (u,0) to a defect at $\left(x_{d},z_{d}\right)$ and back to a receiver element at (v,0), the received signal can be expressed as [21,24],

(17) $$\;\begin{array}{cc} G(u,v,\omega )= & A_{0}\left(\omega \right)\frac{B(u,x_{d},z_{d},\omega )}{d(u,x_{d},z_{d})}\frac{B(v,x_{d},z_{d},\omega )}{d(u,x_{d},z_{d})}S\left(\beta_{i},\beta_{s},\omega \right)\\ & \times e^{-\alpha (d\left(u,x_{d},z_{d}\right)+d\left(v,x_{d},z_{d}\right))}e^{-i\omega \frac{\omega }{c}\left(d\left(u,x_{d},z_{d}\right)+d\left(v,x_{d},z_{d}\right)\right)} \end{array}$$

where, $A_{0}$ is the signal spectrum of the transmitted signal, *B* is the directivity function of an array element [25], α is the measured material attenuation coefficient as shown in Figure 3 [26], $S(\beta_{i},\beta_{s},\omega )$ is the scattering matrix [27] of the defect at the wave incident angle of $\beta_{i}$ and scattering angle of $\beta_{s}$.

The simulated FMC data sets, e(u,v,t), can be obtained using 1-D IFT on G(u,v,ω) with respect to ω. In the simulation, the defects were modelled as the SDHs with 1 mm and 2 mm diameters and their scattering matrices were obtained from [28]. Note that the longitudinal wave speed in aluminium and copper were measured at 6300 m/s and 4600 m/s respectively. In order to make the simulation close to the experimental measurements, the transmitted signal, $A_{0}$, is taken as the back-propagated signal from the back-wall reflection [29], as shown in Figure 2d. This signal has a bandwidth of 0.6–12 MHz at the amplitude of −40 dB of the maximum. Furthermore, note that before forming all images FMC data sets were filtered using a digital filter with a central frequency of 5 MHz (100% fractional bandwidth at 40 dB).

## 4. The Selection of Imaging Parameters

In order to make a fair comparison of the chosen imaging algorithms, reasonable imaging parameters for each imaging algorithm should be found first to ensure good imaging performance is achieved. The TFM imaging algorithm has no free parameters so no optimisation is required. Hence, this section focuses on how to find good imaging parameters for the wavenumber imaging algorithm as well as the time domain and f-k domain PWI algorithms. For the wavenumber imaging algorithm and f-k domain PWI algorithm, the first requirement is to properly define the grid for the image domain. The maximum steering angle and angle increment of plane waves in the time domain and f-k domain PWI algorithms are then discussed and optimised.

### 4.1. Image Grid Definition in the Frequency Domain Algorithms

In the time domain algorithms, the image region and pixel size can be defined arbitrarily, although a smaller pixel sizes lead to smoother images. However, in the frequency domain algorithms, image artefacts produced by Fourier domain wrapping effects [30] must be avoided. Here we assume a Cartesian coordinate system and a rectangular image domain with equally distributed pixels in both *x* and *z* directions. The wavenumbers in the f-k frequency domain, i.e., *k*, $k_{u}$ and $k_{v}$, and those in the image Fourier domain, i.e., $k_{x}$ and $k_{z}$, are then distributed as,

(18) $$\begin{array}{cc} k & =\frac{2\pi }{N_{k}}\frac{f_{s}}{c}\left(-N_{k}/2,\cdots ,0,\cdots ,N_{k}/2-1\right)\\ k_{u} & =\frac{2\pi }{N_{u}}\frac{1}{p}\left(-N_{u}/2,\cdots ,0,\cdots ,N_{u}/2-1\right)\\ k_{v} & =\frac{2\pi }{N_{v}}\frac{1}{p}\left(-N_{v}/2,\cdots ,0,\cdots ,N_{v}/2-1\right)\\ k_{x} & =\frac{2\pi }{N_{x}}\frac{1}{\Delta x}\left(-N_{x}/2,\cdots ,0,\cdots ,N_{x}/2-1\right)\\ k_{z} & =\frac{2\pi }{N_{z}}\frac{1}{\Delta z}\left(-N_{z}/2,\cdots ,0,\cdots ,N_{z}/2-1\right) \end{array}$$

where, $f_{s}$ is the sampling frequency of the time domain data, *p* is the array element pitch, $N_{k}$, $N_{u}$, $N_{v}$, $N_{x}$ and $N_{z}$ are the sizes of the *k*, $k_{u}$, $k_{v}$, $k_{x}$ and $k_{z}$ domains respectively. In practice, $N_{k}$ is the number of points used in the Fast Fourier transform (FFT) of the time domain signals, $N_{u}$ and $N_{v}$ are the numbers of array elements (although zero padding can be used to extend this), $N_{x}$ and $N_{z}$ are the numbers of pixels defined in the reconstructed final image along *x* and *z* directions. From (18), *k* varies from $-(\pi f_{s})/c$ to $(\pi f_{s})/c$, $k_{u}$ and $k_{v}$ from −π/p to π/p determined by the array element pitch, $k_{x}$ and $k_{z}$ span from −π/Δx to π/Δx and −π/Δz to (π/Δz, respectively, determined by the image pixel size.

From (6), $k_{x}$ can by up to $2k_{u}$ and $k_{z}$ to 2k. In order to efficiently use the information from an FMC array data set, the size of the wavenumber space in the image Fourier domain should be greater than that in the f-k domain. This means $k_{x}\geq 2k_{u}$ and $k_{z}\geq 2k$ leading to the requirement for an image pixel size of,

(19) $$\Delta x\leq p/2,\Delta z\leq c/(2f_{s}).$$

In practice, most of the transmitted ultrasonic energy is within a limit bandwidth, hence, to reduce computational cost, Δz in (19) can be taken as the largest frequency in the transmitted bandwidth (i.e., ${f_{bw}}^{max}\leq f_{s}$).

### 4.2. Angle Parameters of the Transmitted Plane Waves in the PWI Algorithms

Here, the choice of maximum steering angle, $\theta_{m}$, and angle increment, Δθ, of the transmitted plane waves in the PWI algorithms is investigated for both the aluminium and copper samples. Note that uniform angular spacing of the plane waves is used and so the angular increment, Δθ, can be calculated from number of plane waves, $N_{p}$, by $\Delta \theta =\left(\theta_{m}^{Optimal}\right)/\left(N_{p}-1\right)$. Plane wave images with $\theta_{m}$ varying from $0^{{}^{\circ}}$ to $80^{{}^{\circ}}$ and at a fixed the angle increment of $\Delta \theta =1^{{}^{\circ}}$ were first generated, and the API (15) and SNR (16) measured from the images.

It is known that when the steering angle of a plane wave is out of range of the geometrical angles between the end array elements and a defect (defined as $\varphi_{1}$ and $\varphi_{2}$ in Figure 1), the emitted wave does not propagate to the defect as a plane wave, hence it generates unwanted artefacts in the image [31]. Hence, it is common for the plane waves with the steering angle greater than the subtended angles to be discarded. This logic leads to a simple route to choosing $\theta_{m}$ which can simply be set separately in the positive and negative direction as, $\varphi_{1}$, $\varphi_{2}$ or to a common value of $\varphi_{c}=\max\left\{\varphi_{1},\varphi_{2}\right\}$. The $\theta_{m}$ that leads to the best API, defined as $\theta_{m}^{API}$, can also be found. Then, using, $\theta_{m}^{Optimal}=\max\left\{\theta_{m}^{API},\varphi_{c}\right\}$, the angle increment was varied from $\Delta \theta =1^{{}^{\circ}}$ to $\Delta \theta =20^{{}^{\circ}}$ with ${\Delta \theta }^{Optimal}$ chosen to be as large as possible without significantly compromising image quality.

#### 4.2.1. Case for the PWI Algorithms on the Aluminium Specimen

**Figure 4:**
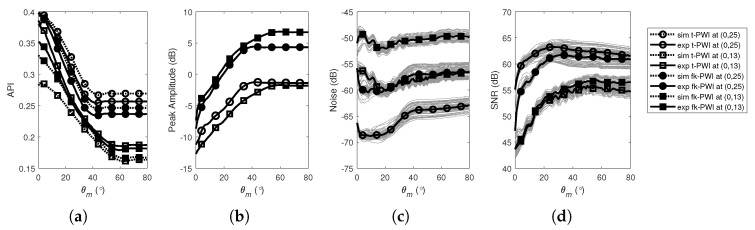
Imaging performance as a function of $\theta_{m}$ from a 1 mm diameter SDH at (0,13) and (0,25) in the aluminium specimen for; (**a**) simulated and experimental API. (**b**) experimental peak amplitude of the defect. (**c**) experimental RMS noise. (**d**) experimental SNR. Note that the amplitudes in (**b**,**c**) are normalized to the peak amplitude in the corresponding back wall image.

Figure 4a compares the API of the time domain plane wave images extracted from the experimental measurements and simulations on a 1 mm diameter SDH at (0,13) and (0,25) in the aluminium specimen. The API decreases with increased $\theta_{m}$ (for $\Delta \theta =1^{{}^{\circ}}$) until a plateau in API performance is reached, i.e., $\theta_{m}^{API}=56^{{}^{\circ}}$ and $40^{{}^{\circ}}$ for the SDHs at (0,13) and (0,25), respectively. Note that these values are close to the geometrical angles as discussed above and shown in Figure 1, i.e., $\varphi_{1}=\varphi_{2}=\tan{}^{-1}\left(\left(N_{e}-1\right)p/2z_{d})\right)=56.8^{{}^{\circ}}$ and $38.4^{{}^{\circ}}$ for the SDH at (0,13) and (0,25), respectively. This confirms that these geometrical angles can be used as a simple way to set the angular range [31]. Figure 4a also shows that the simulations are in good agreement with the experiments (RMS difference between the model and experimental results less than 10% in all cases), meaning that imaging performance and optimisation can be predicted through the simulation.

With regard to the imaging performance of time domain PWI imaging algorithm, Figure 4b shows the experimentally measured peak amplitude from the SDHs as a function of $\theta_{m}$. Peak amplitude is seen to increase with $\theta_{m}$, until it reaches a plateau value at a similar angle to that at which the API plateaued. Figure 4b shows that the experimentally measured noise varies only by a small amount with $\theta_{m}$ and Figure 4d shows the experimental SNR which follows the peak amplitude variation seen in Figure 4b. The plateau point in Figure 4d was measured as $\theta_{m}^{SNR}=37^{{}^{\circ}}$ and $16^{{}^{\circ}}$ for the SDHs at (0,13) and (0,25), respectively. As the SNR in Figure 4d is uniformly high (≥35 dB), minimisation of the API was used here to select the maximum steering angle of the transmitted plane waves, i.e., $\theta_{m}^{Optimal}=\max\left\{\theta_{m}^{API},\varphi_{c}\right\}=\theta_{m}^{API}$.

**Figure 5:**
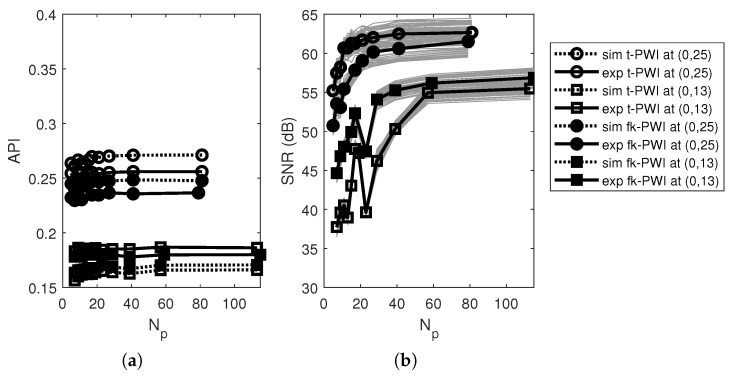
Simulated and experimental imaging performance as a function of $N_{p}$ from a 1 mm diameter SDH at (0,13) and (0,25) in the aluminium specimen for; (**a**) API and (**b**) SNR. Note that in each figure, $\theta_{m}^{Optimal}=\theta_{m}^{API}=56^{{}^{\circ}}$ and $40^{{}^{\circ}}$ for the SDH at (0,13) and (0,25), respectively.

Figure 5a,b show the imaging performance of time domain PWI as a function of number of plane waves, $N_{p}$, when $\theta_{m}^{Optimal}=\theta_{m}^{API}=56^{{}^{\circ}}$ and $40^{{}^{\circ}}$ for the SDH at (0,13) and (0,25), respectively. It can be seen that the API remains almost constant with $N_{p}$ (or Δθ), whereas the SNR increases with increased $N_{p}$ (or decreased Δθ) until a plateau. Hence, there is no optimal value but the choice of $N_{p}$ (or Δθ) is a compromise between detection performance (maximise SNR) and inspection time (minimise the number of firings). The approach taken here is to arbitrarily select the number of plane waves, e.g., $N_{p}=21$.

Table 2 shows the experimentally extracted time domain PWI parameters for 1 mm SDH defect at different locations when $N_{p}=21$. From Table 2, it can be seen that the maximum steering angle, $\theta_{m}^{Optimal}$, is always close to the maximum geometrical angle, $\varphi_{c}=\max\left\{\varphi_{1},\varphi_{2}\right\}$ [31]. This means that the optimal imaging parameters depend on the location of the defect, which is typically unknown. The approach suggested here is to consider defects in a range of locations and select image parameters based on a defect in the worse possible location, which from Table 2 would be at (30,38) with the lowest SNR of 37 dB obtained from $\theta_{m}^{Optimal}=56^{{}^{\circ}}$.

Figure 4a–d also show that the performance of the f-k domain PWI algorithm with angular range follows the same trend as those from the time domain PWI with SNR differing only by ±2 dB. As for the time domain PWI algorithm, the SNR in Figure 4d is high, the imaging performance is optimised by consideration of the API and/or the subtended angles, leading to, $\theta_{m}^{Optimal}=\max\left\{\theta_{m}^{API},\varphi_{c}\right\}=\theta_{m}^{API}=57^{{}^{\circ}}$ and $39^{{}^{\circ}}$ for the SDHs at (0,13) and (0,25), respectively. These optimal parameters are in good agreement with those for the time domain PWI algorithm.

Figure 5a,b also show that the imaging performance of the f-k domain PWI algorithm with angular increment is very similar to the time domain imaging version, with the API having almost no variation with $N_{p}$ (or Δθ) and the SNR increasing with increased $N_{p}$. Repeating the same procedure used to generate Table 2, it is found that the chosen sweep angular range $\theta_{m}^{Optimal}$ from the experimentally measured curves are the same in the time domain and f-k domain PWI algorithms. The worst case is the same as that in the time domain imaging algorithm at (30,38) but with a SNR of 40 dB obtained from $\theta_{m}^{Optimal}=56^{{}^{\circ}}$ and $N_{p}=21$.

#### 4.2.2. Case for the PWI Algorithms on the Copper Specimen

**Figure 6:**
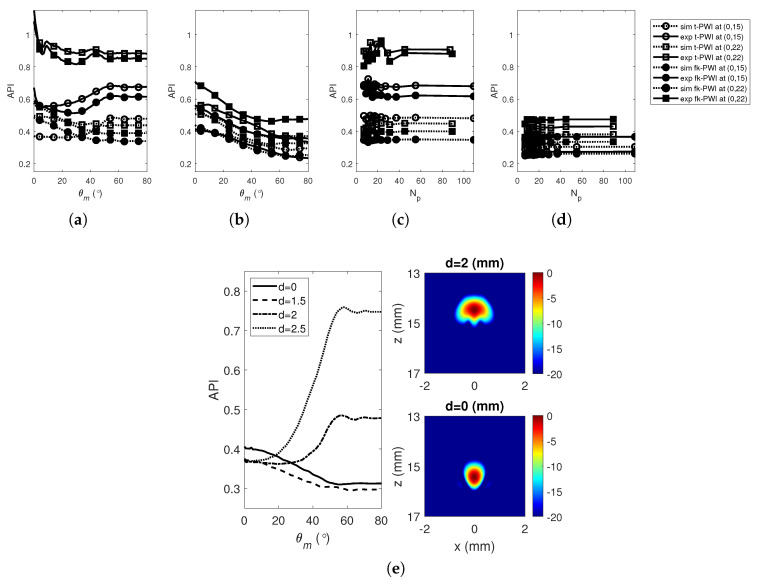
Simulated and experimental imaging performance as a function of $N_{p}$ from a 2 mm diameters SDH at (0,10) and (0,15) in the copper specimen as a function of, (**a**,**b**) $\theta_{m}$ and (**c**,**d**) $N_{p}$. Note that the digital filter (100% fractional −40 dB) used to process the FMC array data sets before forming the images has a centre frequency of; (**a**,**c**) 5 MHz and (**b**,**d**) 1.5 MHz. (**e**) The comparison of the simulated API at 5 MHz from the SDHs with various diameters at (0,15).

Figure 6a,c shows results of time domain PWI and compares the API extracted from the experimental measurements and simulations from a 2 mm diameter SDH in the copper specimen as a function of $\theta_{m}$ and $N_{p}$. As seen in Figure 6a the API varies only slightly with $\theta_{m}$ (for $\Delta \theta =1^{{}^{\circ}}$) which is a distinctly different trend from the aluminium specimen (i.e., Figure 4 and Figure 5). This difference is due to the combined effect of the larger defect geometry used in this case to ensure detection (i.e., a 2 mm SDH) and the multiple scattering from the grain structure which distorts the reflected signals and adds backscattered noise [32]. The effect of defect geometry is explored further in Figure 6e which compares the simulated API as a function of $\theta_{m}$ for SDHs with various diameters. As shown, there is a clear difference between small SDHs (e.g., 1 mm) which are good approximations to point scatterers and larger SDHs (e.g., 2 mm) which result in an extended image and a different trend of API as a function of $\theta_{m}$. Despite these differences, it can also be seen that the $\theta_{m}$ at which the performance plateaus is similar (54${}^{{}^{\circ}}$) across the defects and we note that this angle is close to the geometrical angle (53${}^{{}^{\circ}}$).

The API was also measured from the images after the data sets had been processed using a digital filter with a centre frequency of 1.5 MHz (100% fractional bandwidth at −40 dB) and the results are shown in Figure 6b,d. In this case, with a lower centre frequency, the 2 mm SDHs are now closer to point sctterers and, in line with this the simulation results show a good agreement with the experimentally measured ones. From Figure 6a,c, it is shown that the variation of API with $\theta_{m}$ and $N_{p}$ (or Δθ) is indistinct and hence, unlike the low-noise Aluminium, API is not a good metric to use to select optimal parameters for this noisy material.

**Figure 7:**
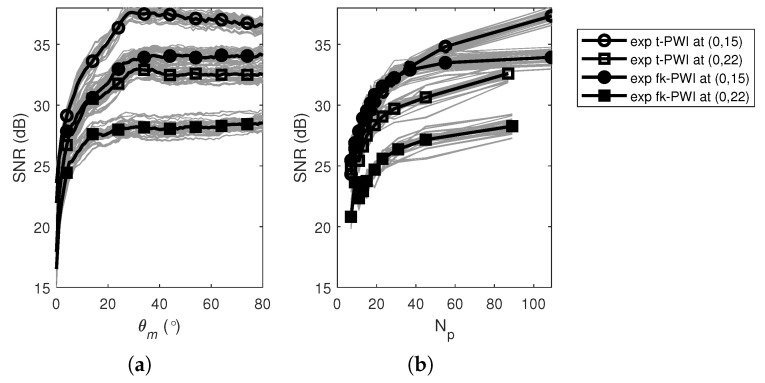
The SNR performance from a 2 mm diameter SDH at (0,10) and (0,15) in the copper specimen as a function of, (**a**) $\theta_{m}$ and (**b**) $N_{p}$. Note that in each figure, $\theta_{m}^{Optimal}=\varphi_{c}=53^{{}^{\circ}}$ and $42^{{}^{\circ}}$ for the SDH at (0,15) and (0,22), respectively.

Figure 7a,b show the SNR of the time domain PWI images from a 2 mm diameter SDH at different depths in the copper specimen, as a function of $\theta_{m}$ and $N_{p}$, respectively. As for the aluminium case, the SNR can be seen to increase with $\theta_{m}$ (for $\Delta \theta =1^{{}^{\circ}}$) to a plateau at around 30${}^{{}^{\circ}}$, which is smaller than the geometric angles of 53${}^{{}^{\circ}}$ and 42${}^{{}^{\circ}}$ from the defect at (0,15) and (0,22), respectively. However, to make choosing imaging parameters simpler, the maximum steering angle was selected as the geometrical angle, $\theta_{m}^{Optimal}=\varphi_{c}$, without compromising the reduction of SNR. Again, following a similar trend to the aluminium, the SNR can be seen to increase as $N_{p}$ increases (or Δθ decreases). As before, there is no optimal value but the choice of $N_{p}$ (or Δθ) is a compromise between detection performance (maximise SNR) and inspection time (minimise the number of firings). For comparison purposes, the approach taken here is to select the number of plane waves as the same as the case for the aluminium specimen, i.e., $N_{p}=21$. Table 3 shows the experimentally extracted time domain PWI parameters for 2 mm SDH defect in the copper specimen at different locations when $N_{p}=21$. It can be seen from Table 3 that the worst optimal case considered was at (25,22) with a SNR of 22 dB for which the PWI parameters were, $\theta_{m}^{Optimal}=64^{{}^{\circ}}$.

## 5. Imaging Performance Comparison

In this section, the computational time from all chosen imaging algorithms is compared. The calculated API and SNR are then used to compare these algorithms in terms of imaging performance. For the scenario where the defect location is unknown and a large inspection area is needed, we fix $\theta_{m}$ to vary from −80${}^{{}^{\circ}}$ to 80${}^{{}^{\circ}}$ and use various $N_{p}$.

### 5.1. Computational Time

The computational costs of the chosen imaging algorithms are made up of the sum of costs from 1-D and 2-D linear interpolations, 2-D and 3-D FFT, as shown in Table 4 [18,23]. One obvious difference seen in Table 4 between the time domain and frequency domain algorithms is the use of the FFT in the imaging reconstruction. In the wavenumber imaging algorithm, a 3-D FFT is first used to process an FMC data set. The 2-D slices of the Fourier transformed data matrix are then taken at each wavenumber sample to calculate the contributions for each image pixel through linear interpolation. Finally, the contributions at each image pixel are summed and the final image is reconstructed through the 2-D inverse Fast Fourier transform (IFFT). In the f-k domain PWI algorithm, the 2-D interpolation and the 2-D FFT are used to speed up imaging.

**Figure 8:**
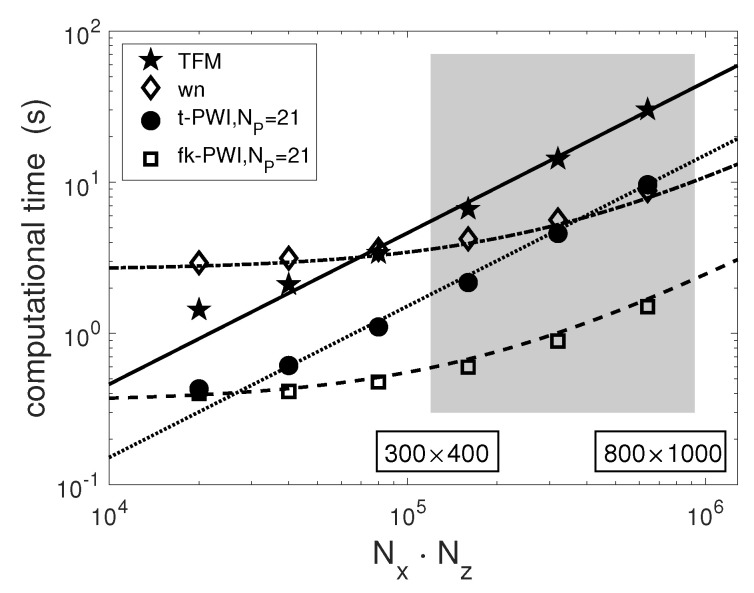
The comparison of the computational time as a function of the number of image pixels from the chosen imaging algorithms. Note that the markers are the actual experimental computation times while the lines are the best fit theoretical line. The grey box indicates the image sizes used in the experiments.

The computation performance of the imaging algorithms inevitably depends to some extent on their implementation. To compare their computation efficiency here, the algorithms were implemented in MATLAB (MathWorks, Natick, MA, USA) on a standard computer with Quad-Core Intel i3-8100 CPU and 8 GB of RAM. The images were generated from the aluminium sample using the chosen imaging algorithms within various image areas ($N_{x}\times N_{z}$). The pixel size was fixed as 0.1 mm, the number of plane waves used for the PWI algorithms is 21 $\left(N_{p}=21\right)$. Figure 8 compares the recorded computational time for the chosen imaging algorithms. Also shown are scaled predictions using the formula shown in Table 4 and the least square fit method, made using the experimentally obtained scale factors $\alpha_{1}=1.1\times 10^{-8},\alpha_{2}=1.6\times 10^{-8},\beta =0.65\times 10^{-8},\gamma =0.5\times 10^{-8}$.

As shown in Figure 8, the computational costs of the TFM and the time domain PWI algorithms increase with the number of image pixels. The wavenumber and the f-k domain imaging algorithms have almost no change in computation time when the number of image pixels is small and then the computation time increases for a large number of image pixels. For larger imaging areas (or larger numbers of pixels), the optimised PWI algorithms have lower computational costs than the TFM and the wavenumber imaging algorithms due to a smaller number of firings ($N_{p}\leq N_{e}$). The efficiency of the frequency domain algorithms can be seen to be superior to the time domain imaging algorithms for large image dimensions. It is also noted that the frequency domain algorithms require a heavy memory load compared with the time domain imaging algorithms. For example, based on the computer resource used here, for imaging an area with $N_{x}\times N_{z}=$ 400 × 300 pixels, the minimum required memory is 26, 1100, 26 and 43 MB for the TFM, the wavenumber, the time domain PWI and f-k domain imaging algorithm, respectively. As is apparent, the largest memory requirement is from the wavenumber imaging algorithm and it is due to a 3D FFT operation in the imaging process [31]. The time domain imaging algorithms can be implemented in a memory efficient fashion by storing only a single time trace (and the final image) in memory at one time. Conversely, the frequency domain imaging algorithms must operate on the entire 3D matrix of echo data simultaneously. It should be noted that the computation efficiency can be increased using GPU parallel processors for both time domain and frequency domain image algorithms.

### 5.2. Image Algorithm Defect Location Performance Comparison for the Aluminium Specimen

**Figure 9:**
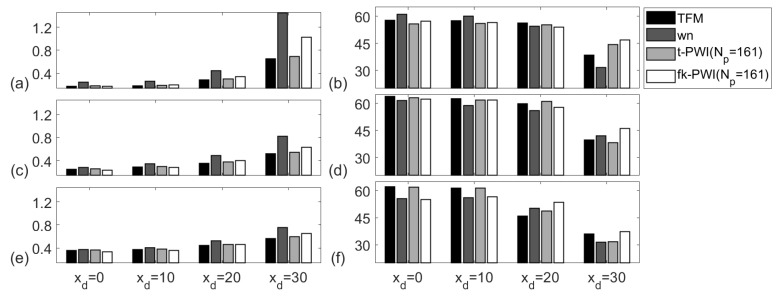
Comparison of imaging performance from the chosen imaging algorithms for the; (**a**,**c**,**e**) API and (**b**,**d**,**f**) SNR (unit is dB). Note that the defect is a 1 mm diameter SDH in the aluminium specimen at a depth of $z_{d}$ = ; (**a**,**b**) 13 mm, (**c**,**d**) 25 mm and (**e**,**f**) 38 mm.

Figure 9 compares the experimentally extracted API and SNR using the chosen imaging algorithms from the 1 mm diameter SDHs in the aluminium specimen for a range of defect locations. Note that $\Delta \theta =1^{{}^{\circ}}\left(N_{p}=161\right)$ is used here as a reference to provide for the best plane wave imaging performance. Due to its large subtended angle, $\varphi_{1}-\varphi_{2}$, the defect nearest to the array centre and the top surface, i.e., at (0,13), has the best API for each imaging algorithm. The TFM and the time domain plane wave images show similar APIs which are between 5% and 55% lower than the other two types of images depending on defect location, with the difference being largest at the extreme, $x_{d}=30$ mm. For each defect located at $x_{d}\leq 20$ mm, the SNR results from all images are similar with variation within 5 dB and this indicates approximately equal performance from all four algorithms. For each imaging algorithm, the SNR at $x_{d}=30$ mm is lower than those at the other locations due to lower directivity of array element and longer ray path.

**Figure 10:**
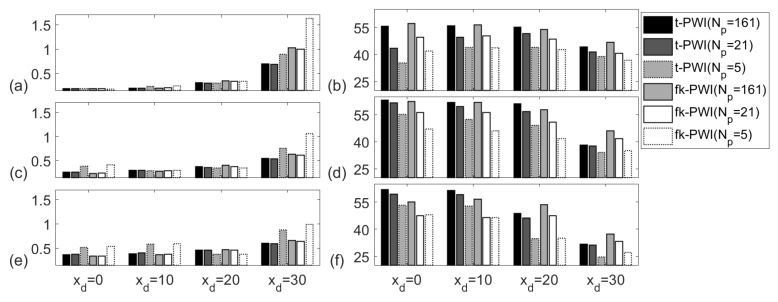
Comparison of imaging performance from the chosen imaging algorithms for the; (**a**,**c**,**e**) API and (**b**,**d**,**f**) SNR (unit is dB). Note that the defect is a 1 mm diameter SDH in the aluminium specimen at a depth of $z_{d}$ = ; (**a**,**b**) 13 mm, (**c**,**d**) 25 mm and (**e**,**f**) 38 mm.

Figure 10 compares the PWI performance for the cases of different numbers of waves which selected according to; (i) $\Delta \theta =1^{{}^{\circ}}$ for best performance $\left(N_{p}=161\right)$; (ii) $N_{p}=21$; (iii) the minimum number of plane waves required to cover all defects $\left(N_{p}=5\right)$. As shown, for the same number of plane waves, the SNRs from time domain plane wave images and f-k domain plane wave images are similar with a variation less than 5 dB. The SNR reduction due to fewer plane waves depends on the defect location. For a fixed defect under a fixed imaging algorithm, the difference of the reduction between the cases of $N_{p}=5$ and $N_{p}=21$ and the cases of $N_{p}=21$ and $N_{p}=161$ varies from 0 dB to 8 dB. This means that the contribution from an extra plane wave to SNR is higher when number of plane waves is low. Furthermore, it should be noted that for low noise materials, even using 5 plane waves can still reach an SNR above 25 dB.

### 5.3. Image Algorithm Defect Location Performance Comparison for the Copper Specimen

**Figure 11:**
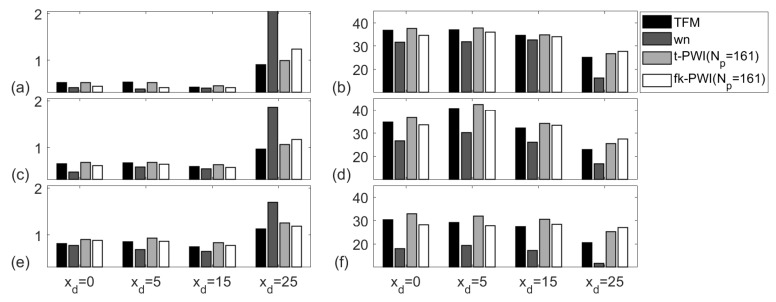
Comparison of imaging performance from the chosen imaging algorithms for the; (**a**,**c**,**e**) API and (**b**,**d**,**f**) SNR (unit is dB). Note that the defect is a 2 mm diameter SDH in the copper specimen at a depth of $z_{d}$ = ; (**a**,**b**) 10 mm, (**c**,**d**) 15 mm and (**e**,**f**) 22 mm.

Figure 11 compares the API and SNR extracted from the chosen imaging algorithms from a 2 mm diameter SDH in the copper specimen. As discussed in Section 4.2.2, the signal can be expected to be distorted due to the multiple scattering from the material grain structure and hence it is difficult to use API to analyse imaging performance. For all defects, the SNR results from the TFM images, the time domain plane wave images and the f-k domain plane wave images show similar SNRs (i.e., within 5 dB) which is at least 5 dB higher than the SNRs from the wavenumber images. This may be due to the number of wavenumbers being 64 (corresponding to $k_{u}$ in Equation (5)) in the wavenumber imaging algorithm, which is less than the number of plane waves (corresponding to θ in Equation (12)), i.e., 161, in the f-k domain plane wave imaging algorithm. Hence noise is better suppressed from more averages in the f-k domain plane wave imaging algorithm than in the wavenumber imaging algorithm.

**Figure 12:**
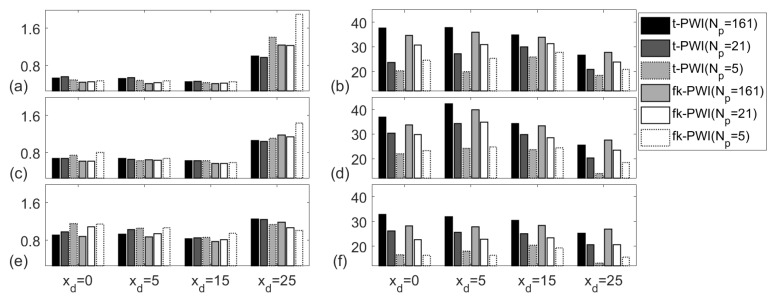
Comparison of imaging performance from the chosen imaging algorithms for the; (**a**,**c**,**e**) API and (**b**,**d**,**f**) SNR (unit is dB). Note that the defect is a 2 mm diameter SDH in the copper specimen at a depth of $z_{d}$ = ; (**a**,**b**) 10 mm, (**c**,**d**) 15 mm and (**e**,**f**) 22 mm.

Figure 12 compares the PWI performance for the cases of different number of waves which selected according to; (i)$N_{p}=161$; (ii) $N_{p}=21$; (iii) $N_{p}=5$. Again, the SNR reduction due to the use of fewer plane waves depends on the defect location. For the copper specimen, more plane waves are needed when compared to the aluminium case to achieve an SNR above 20 dB. Again, the contribution from an extra plane wave to SNR is higher when number of plane waves is low. Note that when $N_{p}=5$, the SNRs from the defects located at either $x_{d}$ = 25 mm or $z_{d}$ = 22 mm are less than 20 dB. This is because of the high attenuation meaning that the defects farthest away from the probe have lower SNRs.

## 6. Conclutions

The imaging performance of the TFM algorithm, the wavenumber imaging algorithm, the time domain and f-k domain PWI imaging algorithms were investigated and compared.

- In order to reduce image artefacts, in the wavenumber and f-k domain PWI imaging algorithms, the pixel size in the array lateral direction is required to be less than a half of the pitch of an array element while that in the depth direction less than a half of the ratio between the wave speed and the highest frequency of the transmitted signals.
- The API performance in the PWI algorithms depends on the subtended angle between an image point and the ends of an array and can be predicted using the proposed simulation model in the single scattering regime. However, when the multiple scattering occurs, the image of the defect is distorted and the SNR is reduced, often making the API unsuitable used for selecting imaging parameters.
- There is no optimal value for the number of plane waves but the choice of number of plane waves is a compromise between detection performance (maximise SNR) and inspection time (minimise the number of firings). When number of plane waves is high, e.g., Np = 161, for low noise material, all chosen imaging algorithms have similar SNR performance, i.e., all SNRs within 5 dB. However, for high noise material, the TFM imaging algorithm, the time domain PWI algorithm and the f-k domain PWI algorithm have similar performance with SNR at least 5 dB higher than that obtained using the wavenumber imaging algorithm.
- 5 plane waves can be used for imaging low noise materials, e.g., aluminium specimens with SNR above 25 dB for a 1 mm SDH defect. However, for imaging materials with high backscattering, e.g., copper specimens, the multiple scattering distorted the API and 21 plane waves were required to achieve SNR greater than 25 dB for a 2 mm SDH defect.

## Figures and Tables

**Table 1:** Specification of the array transducer used in both the experimental measurements and simulations.

Number of elements, $N_{e}$	64
Element width (mm)	0.53
Element pitch, *p* (mm)	0.63
Element length (mm)	15
Central frequency (MHz)	5
Bandwidth(−6 dB) (MHz)	3–7

**Table 2:** The comparison of angle parameters used in the time domain PWI algorithm from the defects at different locations in the aluminium specimen.

Defect Location $\left(x_{d},z_{d}\right)$(mm)	Degree (${}^{{}^{\circ}}$)			
	$\theta_{m}^{Optimal}=\theta_{m}^{API}$	$\varphi_{c}$	$\Delta \theta_{c}$	SNR(dB)
(0,13)	57	57	5.7	47
(10,13)	66	66	5.2	50
(20,13)	76	72	3.6	53
(30,13)	79	75	1.9	42
(0,25)	40	38	3.9	62
(10,25)	52	50	3.6	60
(20,25)	61	58	2.9	59
(30,25)	68	63	2.1	40
(0,38)	30	23	2.8	61
(10,38)	41	38	2.7	60
(20,38)	48	46	2.3	48
(30,38)	56	53	1.9	37

Note that $\Delta \theta_{c}$ is taken at the case of $N_{p}=21$.

**Table 3:** The comparison of angle parameters used in the time domain PWI algorithm from the defects at different locations in the copper specimen.

Defect Location $\left(x_{d},z_{d}\right)$(mm)	Degree (${}^{{}^{\circ}}$)			
	$\theta_{m}^{API}$	$\theta_{m}^{Optimal}=\varphi_{c}$	$\Delta \theta_{c}$	SNR(dB)
(0,10)	37	63	6.3	26
(5,10)	30	66	6.2	29
(15,10)	29	74	5.0	32
(25,10)	62	77	2.5	24
(0,15)	27	53	5.3	31
(5,15)	27	59	5.2	37
(15,15)	24	67	4.3	31
(25,15)	33	72	2.7	24
(0,22)	28	42	4.2	29
(5,22)	20	48	4.1	29
(15,22)	23	58	3.5	28
(25,22)	46	64	2.6	22

Note that $\Delta \theta_{c}$ is taken at the case of $N_{p}=21$.

**Table 4:** Computational complexity of the chosen imaging algorithms.

Operation	Imaging Algorithms			
	TFM	Wavenumber	Time Domain PWI	f-k Domain PWI
1-D interpolation	$\alpha_{1}N_{x}N_{z}N_{e}^{2}$		$\alpha_{1}N_{x}N_{z}N_{e}N_{p}$	
2-D interpolation		$\alpha_{2}N_{x}N_{z}N_{e}$		$\alpha_{2}N_{x}N_{z}N_{e}N_{p}$
3-D FFT		$\beta N_{e}^{2}N_{k}\log_{2}\left(N_{e}^{2}N_{k}\right)$		
2-D FFT				$\;\gamma N_{p}N_{e}N_{k}\log_{2}\left(N_{e}N_{k}\right)$
		$\gamma N_{x}N_{z}\log_{2}\left(N_{x}N_{z}\right)$		$+\gamma N_{x}N_{z}\log_{2}\left(N_{x}N_{z}\right)$

## Supplementary Materials

All data used in this paper are openly available for download from the University of Bristol Research Data Repository at https://doi.org/10.5523/bris.4ecjz523xrb42jtzudg2qlf6p.
